# Breast cancer screening attendance in two Swiss regions dominated by opportunistic or organized screening

**DOI:** 10.1186/s12913-016-1760-4

**Published:** 2016-09-23

**Authors:** Monika Eichholzer, Aline Richard, Sabine Rohrmann, Seraina M. Schmid, Cornelia Leo, Dorothy J. Huang, Uwe Güth

**Affiliations:** 1 Division of Chronic Disease Epidemiology, Epidemiology, Biostatistics and Prevention Institute, University of Zurich, Hirschengraben 84, CH-8001 Zurich, Switzerland; 2 Department of Gynecology & Obstetrics, Spital Grabs, Spitalstrasse 44, CH-9472 Grabs, Switzerland; 3 Breast Center St. Gallen, Rorschacher Strasse 150, CH-9006 St.Gallen, Switzerland; 4 Department of Gynecology and Obstetrics, Kantonsspital Baden AG, Interdisciplinary Breast Centre, CH-5404 Baden, Switzerland; 5 Department of Gynecology and Obstetrics, University Hospital Basel (UHB), Spitalstrasse 21, CH-4031 Basel, Switzerland; 6 Breast Center Zurich, Seefeldstrasse 214, CH-8008 Zurich, Switzerland

**Keywords:** Breast cancer, Mammography screening, Attendance, Opportunistic screening, Organized screening program

## Abstract

**Background:**

In Switzerland, the French-speaking region has an organized breast cancer (BC) screening program; in the German-speaking region, only opportunistic screening until recently had been offered. We evaluated factors associated with attendance to breast cancer screening in these two regions.

**Methods:**

We analyzed the data of 50–69 year-old women (*n* = 2769) from the Swiss Health Survey 2012. Factors of interest included education level, place of residence, nationality, marital status, smoking history, alcohol consumption, physical activity, diet, self-perceived health, history of chronic diseases and mental distress, visits to medical doctors and cervical and colorectal cancer screening. Outcome measures were dichotomized into ≤2 years since most recent mammography versus >2 years or never.

**Results:**

In the German- and French-speaking regions, mammography attendance within the last two years was 34.9 % and 77.8 %, respectively. In the French region, moderate alcohol consumption (adjusted OR 2.01, 95 % CI 1.28–3.15) increased screening attendance. Compared to those with no visit to a physician during the recent year, women in both regions with such visits attended statistically significantly more often BC screening (1–5 times vs. no visit: German (adjusted OR 3.96, 95 % CI 2.58–6.09); French: OR 7.25, 95 % CI 4.04–13.01). Non-attendance to cervical screening had a negative effect in both the German (adjusted OR 0.44, 95 % CI 0.25–0.79) and the French region (adjusted OR 0.57, 95 % CI 0.35–0.91). The same was true for colorectal cancer screening (German (adjusted OR 0.66, 95 % CI 0.52–0.84); French: OR 0.52, 95 % CI 0.33–0.83). No other factor was associated with BC screening and none of the tests of interaction comparing the two regions revealed statistically significant results.

**Conclusion:**

The effect of socio-demographic characteristics, lifestyle, health factors and screening behavior other than mammography on non-attendance to BC screening did not differ between the two regions with mainly opportunistic and organized screening, respectively, and did not explain the large differences in attendance between regions. Other potential explanations such as public promotion of attendance for BC screening, physicians’ recommendations regarding mammography participation or women’s beliefs should be further investigated.

## Background

Opportunistic mammography is a screening tool used by women on their own initiative or following the advice of their gynecologist or general practitioner. Organized breast cancer (BC) screening is a free service offered periodically to asymptomatic women aged e.g. 50–69 years depending on the recommendations of various countries. These screening programs are performed according to guidelines for quality assurance [1, 2]. In Switzerland, organized BC screening programs coexist with opportunistic mammography screening. The Federal Office of Social Insurance decided in 1999 to reimburse biennial mammography screening for women aged 50 years and above through the Swiss compulsory health insurance system, when performed within a quality-assured program [3]. In 2012, the whole French-speaking region of Switzerland was covered by an organized BC screening program. The German-speaking region of Switzerland, on the other hand, started introducing organized screening programs in 2010 in the canton of Thurgovia, 2011 in the cantons of St. Gallen and Grisons, 2013 in the canton of Berne, and 2014 in the canton of Basel-City but not in the rest of the German speaking cantons. Thus, when the 2012 Swiss Health Survey (SHS) was carried out, mainly opportunistic BC screening existed in the German-speaking part of Switzerland [4].

In previous studies, non-attendance to BC screening was associated with lifestyle factors such as smoking [5], lower physical activity [6], and alcohol abstinence [7]. In addition, health-related factors such as poor self-perceived health [7], serious psychological distress [8], and non-attendance to cervical cancer screening were associated with non-attendance [9]. Socio-demographic factors such as lower level of education and lower occupational class [10], living without partner [11, 12], and nationality/migration status [13] may be of importance. The few studies that considered this topic found evidence that factors associated with non-attendance to mammography screening might differ between opportunistic and organized BC screening programs [14, 15].

In a previous analysis of Swiss data on the association between body mass index (BMI) and BC screening attendance, we observed a higher attendance of overweight women overall and of women from the German-speaking region, but not of women from the French-speaking region [16]. Data on factors associated with non-attendance to BC screening according to the type of screening practices are not available in Switzerland and are scarce in other countries.

The aim of the present study was to evaluate further factors that were associated with non-attendance to BC screening according to the main types of screening practices (opportunistic or organized) in two regions of Switzerland. The study sample allowed evaluating various socio-demographic, life-style and health factors in relation to women who do not adhere to mammography screening. In many studies, data on non-attenders are not available. Data from Switzerland is of particular interest, since both types of screening are available simultaneously in a single country, and not-as is usually the case–one screening type being replaced by another.

All analyses were based on data of the Swiss Health Survey (SHS) 2012, and were restricted to 50–69 year-old women, the main target group for BC screening in Switzerland [3].

## Methods

### Study population and design

For the present analysis data of the cross-sectional 2012 SHS conducted by the Swiss Federal Statistical Office [17] (Legal basis: Swiss Statistics, Ordinance of the Conduct of Federal statistical Surveys of 30 June 1993) were used. This survey was comprised of participants randomly selected to represent the Swiss permanent population, i.e. Swiss citizens and foreigners with a legal work permit of both sexes aged 15 years and older. Participants were selected from the resident population living in a private household based on registries of inhabitants. Individuals living in collective households such as nursing homes, military bases, prisons, monasteries, boarding schools, hospitals, hotels etc. were not included. Of a sample of 41,008 individuals, a total of 21,597 agreed to participate in the survey (participation rate 54 %). The level of representativeness of the sample was corrected with appropriate weighting factors from the telephone interview. All individuals were interviewed by telephone (see below “statistical analyses”). Furthermore, all participants were invited to complete a written questionnaire. Only German-, French- or Italian-speaking individuals were included in the survey.

A total of 11,314 women participated in the SHS 2012. Of these, 3,614 women (31.9 %) were 50–69 years old. After excluding women with missing information on BC screening (*n* = 469) and women living in the Italian region of Switzerland (*n* = 247), our sample consisted of 2,898 women. In addition, we excluded women with missing information on demographics (*n* = 18), on lifestyle variables (*n* = 39), on chronic diseases or other screening tests (*n* = 72). Our final dataset consisted of 2,769 participants.

### Measurements

Outcome measure: BC screening status was calculated based on self-reported information on the last BC screening date and then dichotomized in ≤2 years since most recent mammography versus >2 years or never.

Explorative variables: For socio-demographic variables we included educational level (low: compulsory education or less, middle: secondary education vs. high: tertiary education), nationality (Non-Swiss vs. Swiss), area of residence (rural vs. urban), and marital status (single, widowed, divorced, separated, dissolved partner vs. married, registered partnership). Lifestyle factors comprised smoking status (current smoker, former smoker vs. women who have never smoked), hazardous chronic alcohol consumption (≥20 g ethanol daily, <20 g vs. less than once a month or none) [18], physical activity (≥150 min per week vs. less) [19], attention to diet (yes vs. no), and as a potential confounder, body mass index (BMI) categorized into underweight (BMI <18.5 kg/m^2^), normal weight (BMI 18.5–24.9 kg/m^2^), overweight (BMI 25.0–29.9 kg/m^2^), and obesity (BMI ≥30 kg/m^2^). Health-related factors included chronic diseases (self-reported ongoing diseases or health problems lasting for at least 6 months or expected to last a further 6 months), self-perceived health status (fair, poor, very poor vs. very good, good), visits to a physician within the recent year (no, 1–5, 6–10, > 10 times), psychological distress measured by the 5-Item Mental Health Index (MHI-5) (high, moderate, vs. low), cervical cancer screening with the Papanicolaou test (≤3 years since most recent screening vs. >3 years or never), and colorectal cancer screening with colonoscopy (yes [ever] vs. no [never]).

In addition, the question “Who prompted you to attend to this mammography (“own initiative”, “following the recommendation of a medical doctor”, “in the context of a screening program”)” was evaluated.

In the Swiss Health Survey, the entire study sample comprised the German-, French- and Italian-speaking parts of Switzerland, the language regions being defined by the language of the municipality of the study participant’s residence. In the present study, we included the German- and the French-speaking regions and compared them with each other.

### Statistical analyses

We evaluated potential differences of attendance to BC screening in the German-speaking region with mainly opportunistic screening and in the French-speaking region of Switzerland with population based programs depending on socio-demographics, lifestyle and health-related factors as well as non-attendance to screening other than mammography.

In our analyses, we used two logistic regression models. Model 1 was unadjusted and model 2 was adjusted for all other demographic, lifestyle and health factors as shown in Table 1, with the exception of cervical and colorectal cancer screening.

**Table 1:** Baseline characteristics (socio-demographic, lifestyle/health factors, and screening behavior other than mammography) in 50–69 year-old women: Swiss Health Survey 2012^a^

		German region	French region
Women, n		1938	831
Age, mean		58.5	59.1
		*%*	*%*
Educational level	High	19.4	24.9
	Middle	66.5	56.1
	Low	14.1	19.0
Nationality	Swiss	89.9	84.7
	Non-Swiss	10.1	15.3
Place of residence	Urban	71.1	74.5
	Rural	28.9	25.5
Marital status	married/registered partnership	66.0	59.4
	Single, divorced/dissolved partnership, separated, widowed	34.0	40.6
BMI	Underweight (BMI < 18.5 kg/m^2^)	3.6	4.0
	Normal weight (BMI 18.5–24.9 kg/m^2^)	55.8	58.1
	Overweight (BMI 25–29.9 kg/m^2^)	27.5	27.8
	Obesity (BMI ≥ 30 kg/m^2^)	13.1	10.2
Smoking status	Never smoker	50.9	44.1
	Ex-smoker	25.7	30.5
	Current smoker	23.4	25.4
Alcohol	Never	27.6	33.6
	<20 g ethanol per day	68.5	60.0
	≥20 g ethanol per day	4.0	6.4
Physical activity	≥150 min. per week	76.2	60.3
	<150 min. per week	23.7	39.7
Attention to diet	Yes	86.6	64.8
	No	13.4	35.2
Self-perceived health	Good, very good	81.1	71.4
	Fair, poor, very poor	18.9	28.6
Chronic disease^b^	No	57.0	59.5
	Yes	43.0	40.5
Psychological distress	Low	82.8	73.3
	Moderate	12.0	18.3
	High	4.9	8.4
Visits to a physician within the recent year	no	16.5	10.7
	1 to 5 times	62.8	66.4
	6 to 10 times	12.6	13.9
	>10 times	7.8	9.1
Cervical cancer screening	Yes	90.8	78.3
	No	9.2	21.7
Colorectal cancer screening	Yes	36.0	35.9
	No	64.0	64.1

^a^ all proportions are weighted according the Swiss general population, except n

^b^ self-reported ongoing diseases or health problems lasting for at least 6 months or expected to last further 6 months

Tests for interaction were done using the cross-product terms of each of the exposure variables (demographics, lifestyle and health factors) with the dichotomized region of Switzerland (German vs. French); this interaction term was evaluated using the Wald test. *P*-values <0.05 were considered statistically significant.

Sampling weights of the telephone interviews were provided by the Swiss Federal Statistical Office (SFSO) and applied to the data of the present analyses to calculate descriptive characteristics (percentages) and to conduct logistic regression analyses. The sampling weights include a comparison with the permanent 2012 Swiss population with regard to sex, age, geographic region and nationality (Swiss vs. others). All calculations and analyses were performed with STATA/SE, version 13 (StataCorp, College Station, Texas).

## Results

Table 1 summarizes socio-demographic characteristics, lifestyle and health factors, and screening behavior other than mammography of the 1938 eligible women of the German-speaking region and of the 831 women of the French-speaking region of Switzerland participating in the 2012 SHS. Of the German-speaking women, higher percentages reached the recommendations for physical activity (76.2 vs. 60.3 %), paid attention to their diet (86.6 vs. 64.8 %), declared themselves to be in good health (81.1 vs. 71.4 %) and had a cervical cancer screening test (90.8 vs. 78.3 %) compared to French-speaking women.

Table 2 shows that in the German-speaking region of Switzerland with its mainly opportunistic BC screening, 28.8 % of the 50–69 year-old women never had a BC screening examination, 34.9 % had one within the past two years, and 36.3 % had a mammography more than two years ago. The corresponding values in the French-speaking region, with population based BC screening programs, were 7.3 %, 77.8 %, and 15.0 %. Of the women who attended BC screening in the German-speaking region, 16.8 % did it on their own initiative, 73.1 % followed the recommendation of a medical doctor, and 10.1 % in the context of a screening program. In the French-speaking region, the corresponding values were 8.5 %, 36.5 % and 55.1 %, respectively.

**Table 2:** Attendance to mammography in the German- and French-speaking regions of Switzerland in 50–69 years-old women: Swiss Health Survey 2012^a^

		German region	French region
Women, n		1938	831
		*%*	*%*
Mammography	Never	28.8	7.3
	Within the last 2 years	34.9	77.8
	More than 2 years ago	36.3	15.0
Who prompted you to attend to this mammography?			
	Own initiative	16.8	8.5
	Following the recommendation of a medical doctor	73.1	36.5
	In the context of a screening program	10.1	55.1

^a^all proportions are weighted according to the Swiss general population, except n

Associations between socio-demographic characteristics, lifestyle and health-related factors, screening behavior other than mammography, and BC screening attendance are presented separately for the two regions in Table 3. In the German-speaking region, BC screening attendance was significantly less common among women not adhering to cervical cancer screening (adjusted OR 0.44, 95 % CI 0.25–0.79) and colonoscopy (adjusted OR 0.66, 95 % CI 0.52–0.84). In the French-speaking region, moderate alcohol consumption (adjusted OR 2.01, 95 % CI 1.28–3.15) was associated with significantly increased attendance to BC screening. As in the German-speaking region, low attendance to cervical screening and colonoscopy was statistically significantly associated with a lower attendance to BC screening (adjusted OR 0.57, 95 % CI 0.35–0.91 and OR 0.52, 95 % CI 0.33–0.83). Compared to those with no visit to a physician during the recent year, women in both regions with such visits attended statistically significantly more often BC screening (1–5 times vs. no visit: German (OR 3.96, 95 % CI 2.58–6.09); French: OR 7.25, 95 % CI 4.04–13.01). With the exception of the thus far mentioned associations with BC screening, none of the other socio-demographic and lifestyle factors (education, living in urban/rural areas, nationality, marital status, smoking status, attention to diet and physical activity, and health issues, characterized by self-perceived health, chronic disease status and mental distress) was statistically significantly associated with mammography use, in both the German and the French-speaking regions. Furthermore, none of the tests of interaction comparing each single explorative variable with the two regions revealed a statistically significant result.

**Table 3:** Associations of mammography attendance^a^ with socio-demographic, lifestyle/health factors, and screening behavior other than mammography in 50-69 years-old women: Swiss Health Survey 2012^b^

		German region				French region				
		unadjusted		multivariable adjusted^c^		unadjusted		multivariable adjusted^c^		
		*OR*	*95 % CI*	*OR*	*95 % CI*	*OR*	*95 % CI*	*OR*	*95 % CI*	*P-Interaction*
Educational level	High	1		1		1		1		
	Middle	1.27	(0.94-1.72)	1.17	(0.85-1.60)	1.02	(0.63-1.63)	0.85	(0.50-1.44)	
	Low	1.16	(0.75-1.80)	1.08	(0.68-1.71)	0.86	(0.48-1.54)	0.60	(0.32-1.12)	0.208
Nationality	Swiss	1		1		1		1		
	Non-Swiss	0.95	(0.59-1.51)	0.91	(0.57-1.46)	1.32	(0.70-2.49)	1.41	(0.73-2.73)	0.360
Place of residence	Urban	1		1		1		1		
	Rural	1.14	(0.88-1.47)	1.16	(0.88-1.51)	0.86	(0.57-1.29)	0.96	(0.60-1.52)	0.255
Marital status	married / registered partnership	1		1		1		1		
	Single, divorced / dissolved partnership, separated, widowed	0.78	(0.61-1.01)	0.82	(0.63-1.06)	0.77	(0.53-1.13)	0.76	(0.51-1.14)	0.808
Smoking status	Never smokers	1		1		1		1		
	Ex-smoker	1.33	(1.01-1.74)	1.28	(0.96-1.70)	1.17	(0.74-1.86)	0.97	(0.59-1.59)	
	Current smokers	0.87	(0.64-1.17)	0.92	(0.67-1.26)	0.76	(0.49-1.18)	0.73	(0.45-1.18)	0.399
Alcohol	Never	1		1		1		1		
	< 20 g ethanol per day	1.19	(0.92-1.55)	1.21	(0.92-1.59)	1.86	(1.24-2.77)	2.01	(1.28-3.15)	
	≥ 20 g ethanol per day	0.86	(0.49-1.52)	0.94	(0.53-1.67)	1.00	(0.50-2.01)	0.98	(0.45-2.11)	0.270
Physical activity	≥ 150 min. per week	1		1		1		1		
	< 150 min. per week	0.88	(0.67-1.15)	0.91	(0.68-1.21)	0.79	(0.54-1.14)	0.73	(0.49-1.09)	0.355
Attention to diet	Yes	1		1		1		1		
	No	0.86	(0.60-1.22)	1.01	(0.70-1.44)	0.81	(0.55-1.19)	1.04	(0.69-1.56)	0.565
Self-perceived health	Good, very good	1		1		1		1		
	Fair, poor, very poor	1.25	(0.92-1.70)	1.08	(0.75-1.55)	1.81	(1.17-2.80)	1.66	(0.98-2.81)	0.205
Chronic disease^d^	No	1		1		1		1		
	Yes	1.24	(0.99-1.57)	0.90	(0.69-1.17)	1.60	(1.09-2.36)	1.13	(0.72-1.77)	0.390
Psychological distress	Low	1		1		1		1		
	Moderate	1.11	(0.78-1.59)	1.03	(0.72-1.47)	1.32	(0.83-2.10)	1.16	(0.69-1.94)	
	High	0.85	(0.47-1.53)	0.70	(0.38-1.30)	1.36	(0.69-2.69)	1.33	(0.61-2.86)	0.219
Visits to a physician within the recent year	no	1		1		1		1		
	1-5 times	4.02	(2.64-6.11)	3.96	(2.58-6.09)	7.54	(4.31-13.17)	7.25	(4.04-13.01)	
	6 to 10 times	4.31	(2.57-7.21)	4.26	(2.49-7.30)	6.71	(3.35-13.46)	5.51	(2.60-11.68)	
	> 10 times	6.43	(3.72-11.13)	7.03	(3.88-12.73)	6.14	(2.69-13.98)	6.07	(2.54-14.52)	0.976
Cervical cancer screening	Yes	1		1		1		1		
	No	0.38	(0.21-0.69)	0.44	(0.25-0.79)	0.44	(0.29-0.67)	0.57	(0.35-0.91)	0.786
Colorectal cancer screening	Yes	1		1		1		1		
	No	0.56	(0.44-0.71)	0.66	(0.52-0.84)	0.43	(0.28-0.65)	0.52	(0.33-0.83)	0.235

^a^mammography screening adherence defined by participation within the last 2 years

^b^weighted according the Swiss general population

^c^adjusted for all factors shown in Table 1, except cervical cancer and colonoscopy screening

^d^self-reported ongoing disease or health problem lasting for at least 6 months or expected to last further 6 months

## Discussion

The participation rate of 77.8 % in the French-speaking region of Switzerland lies within the range of other European countries’ screening uptake of 55 to 90 % [20]. The much lower mammography attendance in the German-speaking region of Switzerland (34.9 %) confirms previous observations that women are more likely to adhere to BC screening in countries with screening programs than in those with opportunistic screening [15, 21]. Factors associated with non-attendance to BC screening might differ between opportunistic and organized BC screening programs [12, 14, 15] and the question arises whether organized screening programs “rescue” distinct subgroups of women who fall through the opportunistic screening net? In the present study, we did not find any socio-demographic, lifestyle or health factors, which could explain the large difference in BC screening attendance between the French- and the German-speaking regions of Switzerland. None of the interaction terms were statistically significant.

In contrast to previous observations demonstrating that a socio-economic gradient is evident in BC screening attendance in opportunistic but not in population-based programs [14], in the present study, neither attendance to opportunistic nor organized BC screening were associated with educational level, nationality, living in urban or rural areas or marital status. Previous studies observed that women with higher educational levels reported greater screening participation [11, 22], although not consistently so [23–25]. It should be noted that in Switzerland, all residents are obliged by law to have a health insurance, thus, BC screening costs are basically covered [26]. This might explain why education, as potential indicator of the women’s socioeconomic status, was not associated with BC screening attendance in the present study. In many studies [11, 27, 28], marital status was associated with higher attendance to BC screening. Partners seem to encourage each other towards health conscious behaviors [29]. A survey carried out in Geneva, Switzerland, found that men had more favorable attitudes towards BC screening than women [30]. Living with a partner, but not being married or registered (registration is in Switzerland only an option for couples of the same sex), is considered in the Swiss Health Survey as being single. This may explain why we did not observe an association between marital status and BC screening attendance.

In our study, additional socio-demographic factors such as nationality and living in a rural or urban area also had no effect on screening attendance rates in both regions. Lower screening participation in urban areas has previously been reported from various European countries [25, 31, 32]. The different demographic composition of urban and rural areas in relation to migrants, education and economic level of the inhabitants may contribute to such differences [32]. Participation to the Swiss Health Survey was limited to German-, French- or Italian-speaking individuals. Thus, the group of immigrants in the present study is restricted to those who speak German, French or Italian. It does therefore not reflect reality in Switzerland in relation to the various immigrant groups living in this country, and may have excluded minorities prone to non-attendance of BC screening [11, 33]. Using more detailed data of the 2002 SHS, attendance to BC screening was higher in Swiss women than in women from Italy, former Yugoslavia, Portugal and Spain living in Switzerland [34].

In relation to lifestyle factors, represented by smoking habits, alcohol intake, physical activity and attention to diet, only moderate alcohol consumption in the French-speaking region showed an association with BC screening; however, no statistically significant interactions between the two regions were observed. There is some evidence from other studies that former smokers have a higher attendance to BC screening [35, 36], but many previous studies failed to distinguish between former smokers and those who had never smoked. As Vander Weg [36] pointed out, former smokers may see themselves with a higher cancer risk due to their smoking habits in the past and, therefore, may be more motivated to adhere to screening recommendations than non-smokers. The findings of lower rates of mammography among current smokers, which have been rather consistently shown in other studies [7, 35, 36], were not confirmed in the present study and in another survey [22].

In our study, alcohol consumption had no effect on the rates of having had BC screening with the exception of higher BC screening attendance of women from the French-speaking region who reported drinking moderate amounts of alcohol. Results from other studies [7, 28] indicate a J-shaped association between alcohol consumption and BC screening use, where both abstainers and heavy drinkers have lower attendance than women drinking modest amounts. The absence of an association between high and low alcohol intake and mammography attendance in our study may be due to information bias and a lower participation rate of heavy drinkers in research studies [7, 37].

In relation to physical activity, our findings are not in line with the results of previous studies, in which non-attendance to BC screening was associated with lower leisure time physical activity [7]. There are too few studies analyzing the association between nutritional habits and BC screening attendance to come to a meaningful conclusion [38, 39].

Impaired overall health and more frequent contact with medical doctors, respectively, on the other hand, may have implications for BC screening. Studies have shown that factors such as diabetes, cognitive decline, and depression might negatively affect the receipt of cancer screening [8, 22, 28]. The presence of several comorbid conditions might increase the opportunity of receiving cancer screening or diagnosis, however, because of more frequent contact with medical doctors. This could be an explanation for the positive association observed between visits to a physician (compared to none) and higher attendance to BC screening in the present and other studies [40].

Schumacher et al. [22] observed in their Education and Research Towards Health study that those women who had had other screening tests (cervical and colorectal cancer screening) were much more likely to have received mammography in the past two years. This was true for opportunistic and organized screening, as in the present study. Similar results were seen for organized BC screening in a study carried out in Geneva [41]. Individuals who participate in multiple cancer screenings may be more health-conscious, and/or more knowledgeable about cancer screening tests than those who do not [9, 28, 42]. Those with low attendance to screening tests, on the other hand, may be more critical about the benefits of the screening, including anxiety related to false positive results [43] and inaccurate knowledge and negative mammography beliefs such as that mammography may be harmful, is only needed when symptoms are present, is not necessary, is painful, etc. [42].

### Study strengths and limitations

One strength of our study is the large representative sample of individuals 15 years and older living in Switzerland, allowing for limitation of our analyses to women aged 50–69 years. This study also has several limitations. It is a cross-sectional survey, thus, causality cannot be inferred. In addition, data were self-reported. Women, for example, tend to overestimate their attendance to cancer screening according to the recommendations [44]. This could result in reporting and/or recall biases. Furthermore, 46 % of eligible subjects did not participate in the SHS survey. However, we hypothesize that with respect to mammography attendance rates our survey cohort represents the general population very well because the attendance to the mammography screening programs in the French-speaking cantons were similar to the official data of Swiss Cancer Screening in 2011 (51 %) [4]. In addition, the use of weighting factors allowed for the extrapolation of the results in relation to age, gender, region and nationality from the sample to the total population living in Switzerland [17]. Furthermore, attendance to opportunistic or organized screening was not clear-cut in the present study, i.e. in the German- and in the French-speaking regions, 10.1 % and 55.1 %, respectively, had a mammography in the context of a screening program, i.e. in the German-speaking region 16.8 % attended mammography on their own initiative, 73.1 % followed the advice of a medical doctor (French-speaking region: 8.5 % and 36.5 %, respectively). Women with a personal or family history of breast diseases might undergo regular radiologic breast imaging and thus, do not attend a screening BC program. In particular, women who had mammography in previous years in private radiology practices might prefer to continue these examinations in this trusted setting.

Finally, other factors that may be significant determinants of BC screening utilization have not been recorded in the current study [20, 21, 42, 45], such as promotion of BC screening by the government, cancer leagues, medical organizations and lay press. Furthermore, personal or family BC history, knowledge and beliefs in the benefits of screening, and attitude of gynecologists and other medical doctors [46, 47] towards BC screening may be of importance.

## Conclusion

The effect of socio-demographic, lifestyle and health factors and screening behavior other than mammography on non-attendance to BC screening did not differ between opportunistic and organized screening in Switzerland and did not explain the large differences in BC screening attendance between the two regions. Physicians’ recommendations regarding mammography and/or invitations to attend organized screening should be investigated as potential explanations for the lower attendance rate in the German-speaking region. The negative effect of non-attendance to cervical and colorectal cancer screening on BC screening in both regions may reflect women’s negative beliefs and anxiety. This should also be further investigated.

## Abbreviations

BC: Breast cancer
BMI: Body mass index
MHI-5: 5-Item Mental Health Index
SFSO: Swiss Federal Statistical Office
SHS: Swiss Health Survey
